# Silencing of LINC01963 enhances the chemosensitivity of prostate cancer cells to docetaxel by targeting the miR-216b-5p/TrkB axis

**DOI:** 10.1038/s41374-022-00736-4

**Published:** 2022-02-12

**Authors:** Zengshu Xing, Sailian Li, Jiansheng Xing, Gang Yu, Guoren Wang, Zhenxiang Liu

**Affiliations:** 1 Department of Urology, Affiliated Haikou Hospital of Xiangya Medical College, Central South University, 570208 Haikou, China; 2 Department of Gastroenterology, Affiliated Haikou Hospital of Xiangya Medical College, Central South University, 570208 Haikou, China

**Keywords:** Prostate cancer, Cancer therapeutic resistance

## Abstract

Docetaxel (DTX) treatment effectively prolongs the overall survival of patients with prostate cancer. However, most patients eventually develop resistance to chemotherapy and experience tumor progression or even death. Long noncoding RNAs (lncRNAs) affect docetaxel chemosensitivity. However, the biological role and regulatory mechanisms of lncRNAs in docetaxel-resistant prostate cancer remain unclear. Differences in lncRNAs were evaluated by lncRNA sequencing and evaluated using quantitative real-time polymerase chain reaction, and TrkB expression was measured through western blot analysis. Proliferation was measured using the MTS, while apoptosis and cell cycle were measured using flow cytometry. In addition, migration and invasion were measured using transwell assays. Forty-eight female BALB/c nude mice were used for subcutaneous tumorigenicity and lung metastasis assays. We found that LINC01963 was overexpressed in the PC3-DR cells. LINC01963 silencing enhanced the chemosensitivity of PC3-DR to docetaxel and inhibited tumorigenicity and lung metastasis, while LINC01963 overexpression enhanced the chemoresistance of PC3 cells to docetaxel. It was found that LINC01963 bind to miR-216b-5p. The miR-216b-5p inhibitor reversed the suppressive effect of sh-LINC01963 on PC3-DR cell proliferation, migration, and invasion. Furthermore, miR-216b-5p can bind to the 3′-UTR of NTRK2 and inhibit TrkB protein levels. TrkB enhances docetaxel resistance in prostate cancer and reverses the effects of LINC01963 silencing and miR-216b-5p overexpression. In conclusion, silencing LINC01963 inhibited TrkB protein level to enhance the chemosensitivity of PC3-DR to docetaxel by means of competitively binding to miR-216b-5p. This study illustrates that LINC01963 is a novel therapeutic target for treating prostate cancer patients with DTX resistance.

## Abstract (web summary)

The authors show that LINC01963 is overexpressed in the PC3-DR cells and that LINC01963 silencing enhances the chemosensitivity of PC3-DR to docetaxel and inhibits tumorigenicity and lung metastasis. Silencing of LINC01963 ireduces TrkB protein levels to enhance the chemosensitivity of cells to docetaxel by competitively binding to miR-216b-5p.

## Introduction

Prostate cancer is a malignant tumor with the highest incidence in men in Europe and the United States^1^. In China, its incidence is increasing yearly. It is estimated that there were 72,000 new cases of prostate cancer and ~37,000 deaths in China in 2015^2^. Most patients are first diagnosed after the cancer has metastasized. Currently, endocrine therapy is the first-line treatment for patients with advanced metastatic prostate cancer^3^. However, after endocrine therapy with a median time of 18–24 months, almost all patients progress to castration-resistant prostate cancer (CRPC)^4^. Once a patient enters the CRPC stage, the prognosis is generally poor. Docetaxel (DTX) treatment effectively prolonged the overall survival of patients with CRPC. However, most patients eventually develop resistance to chemotherapy and experience tumor progression or even death^5^. Therefore, exploring the molecular mechanisms underlying CRPC-DTX resistance has become particularly urgent.

Long noncoding RNA (lncRNA), especially androgen receptor-related lncRNA, had the importantly clinical utility in the tumor progression, diagnosis and prognosis, and as therapeutic targets of prostate cancer^6–8^. LncRNA NEAT1 promoted bone metastasis via miR-205-5p in prostate cancer^9^. CCAT1 promotes tumor development and progression via miR-490-3p/FRAT1 axis^10^. Increasing evidence suggests that lncRNAs play a key role in the chemoresistance of prostate cancer^11^. Previous studies have found that HOTTIP, PCBP1-AS1, and HOTAIR enhance cisplatin, enzalutamide, and DTX resistance in prostate cancer, respectively^12–14^. In prostate cancer, MALAT1 expression is highly expressed in DTX-resistant tissues and promotes enhanced DTX chemoresistance by regulating the miR-145-5p/AKAP12 axis^15^. In addition, DANCR enhanced DTX resistance in prostate cancer by targeting miR-34a-5p/JAG1 axis^16^. However, the role of lncRNAs in DTX resistance in prostate cancer remains poorly understood. Thus, new research is urgently needed to elucidate the potential mechanism of DTX resistance in prostate cancer.

In this study, we uncovered the effect of DTX resistance on lncRNA expression in PC3 cells using lncRNA sequencing. Next, we demonstrated the effect of LINC01963 on DTX resistance in prostate cancer, in addition to the potential mechanism underlying the effect of LINC01963 on this resistance. This research confirmed that LINC01963 affects DTX resistance in prostate cancer, providing a potential therapeutic target for these patients.

## Materials and methods

### Cell culture and DTX treatment

The human prostate cancer cell line PC3 was cultured in Dulbecco’s modified Eagle’s medium (DMEM; Gibco, Grand Island, NY, USA) with 10% fetal bovine serum (FBS) and maintained in an incubator containing 5% CO_2_ at 37 °C. DTX resistance was induced in the prostate cancer cell line PC3 (PC3-DR) by treating cells with DTX suspended in dimethyl sulfoxide (Sigma, St. Louis, MO, USA) as previously described^17^. Briefly, PC3 cells were treated with DTX at 4 and 8 nmol/L for 48 h for 5 cycles. After treatment, the surviving cells were reseeded into new DMEM for 2–3 weeks. PC3 cells were treated with DTX at 8 and 12 nmol/L for 48 h for 12 cycles. The surviving cells were then reseeded into DMEM for 2–3 weeks. Cells were continuously maintained in DTX, with treatments beginning at 10 nmol/L and gradually increasing by 5 nmol/L increments to a final dose of 50 nmol/L. DMEM containing DTX was changed every 2–3 days. PC3-DR cells were maintained for 1 month with 40 nM DTX.

### MTS

The proliferation of PC3 and PC3-DR cells was analyzed using the MTS assay (Promega, St. Louis, MO, USA). PC3 and PC3-DR cells were cultured in DMEM supplemented with DTX. After 24, 48, and 72 h of culture, 10 μL CellTiter 96 AQueous One Solution Cell Proliferation Assay was added and incubated for 4 h. The OD values were measured at 570 nm using a Multiskan Mk3 microplate reader (Thermo Fisher). The half maximal inhibitory concentration (IC50) value is the concentration of each compound that inhibits cell proliferation by 50% under the experimental conditions and is the average of eight replicate experiments.

### LncRNA sequencing

Total RNA from PC3 and PC3-DR cells with three parallel samples from each group was extracted using TRIzol (Invitrogen, Carlsbad, CA, USA). The purity, concentration, and integrity of the total RNA were evaluated using an Agilent Bioanalyzer 2100 (Agilent Technologies, Santa Clara, CA, USA). LncRNA sequencing was performed using the Illumina HiSeqTM 4000 and bioinformatics analysis^18^.

### Quantitative real-time polymerase chain reaction (qRT-PCR)

Total RNA was isolated using TRIzol^®^ reagent (Invitrogen) and dissolved in 15 μL of DEPC-treated water. The RNA concentration was reverse-transcribed using M-MLV (Promega) and incubated at 42 °C for 60 min. RT-qPCR was performed with the SYBR Green PCR Master Mix (TOYOBO) using the ABI PRISM^®^ 7500 Sequence Detection System (Foster City, CA, USA). Relative expression levels were determined using the 2^−ΔΔct^ method^19^. GAPDH levels were used as internal controls for the lncRNAs and mRNAs. U6 levels were used as internal controls for miRNAs.

### Constructed stable cell lines

Three short hairpin RNAs targeting LINC01963 (sh-LINC01963) and the LINC01963 full-length gene, located at Chro2:216217045-216220192 (+), were packaged into a lentivirus to construct lentiviruses expressing sh-LINC01963 or full-length LINC01963, termed sh-LINC01963 or ov-LINC01963 lentivirus (Genepharma, Shanghai, China). PC3 and PC3-DR cells were infected with sh-LINC01963 and ov-LINC01963 lentivirus and empty control lentivirus (NC) to construct PC3 and PC3-DR stable cell lines. Stable cell lines were collected and resuspended for further analyses.

### Cell cycle and apoptosis analysis

After DTX treatment for 48 h, the cell cycle and apoptosis of PC3 and PC3-DR cells were detected using cell cycle and apoptosis detection kits (Keygen, Nanjing, China), respectively. Samples were analyzed using a BD Calibur flow cytometer (BD Biosciences, Franklin Lakes, NJ, USA).

### Transwell migration/invasion assay

For migration and invasion assays, polycarbonate filters (8 μm pore size, Corning, Corning, NY, USA) uncoated or precoated with Matrigel (BD Biosciences, San Jose, CA, USA) were used. PC3 and PC3-DR cells (1 × 10^5^) were cultured in 300 μL medium containing 0.1% FBS and seeded in the upper chamber. Then, 600 μL of medium supplemented with 10% FBS was added to the lower chamber. After 48 h of incubation, the cells that migrated and adhered to the lower chamber were fixed in 4% paraformaldehyde for 20 min, stained with hematoxylin, and counted under an upright microscope (five fields per chamber).

### Wound healing assay

PC3 and PC3-DR cells (1 × 10^5^ cells per well) were inoculated into a six-well plate overnight. Next, a pipette was used to scratch a vertical line in the cell plate and remove the detached cells. The cells were then treated with DTX for 48 h, observed, and photographed. Each experiment was conducted three times to obtain the average values.

### Tumor xenograft and lung metastasis

The animal experiments were approved by the Affiliated Haikou Hospital of Xiangya Medical College, Central South University. Forty-eight male BALB/c nude mice were obtained from the Guangdong Medical Laboratory Animal Center (SCXK(Yue)2013-0002, Guangzhou, China) and were maintained under SPF conditions (25 °C and 12 h light/dark cycle) for feeding, and all animals were raised under the same conditions to minimize potential confounders. Mice were anesthetized using an anesthetic machine with MAC 1.6% isoflurane. After the experiments, the rats were sacrificed by intraperitoneal injection of 3% pentobarbital sodium (140 mg/kg) and cervical dislocation. In addition, if the mice showed rapid weight loss (>20% body weight loss), were becoming cachectic, self-harming, biting, displaying aggressiveness, difficulty eating, drinking, or moving around freely, which were considered humane endpoints to kill, they were euthanized. All mice were included in this study. For subcutaneous tumorigenicity analysis, 5 × 10^6^ cells were subcutaneously injected into the right flank of 6-week-old female BALB/c nude mice. A total of 24 BALB/c nude mice were divided into four groups by the sorting randomization method (*n* = 6): PC3-DR, PC3-DR + NC, PC3-DR + ov-LINC01963, and PC3-DR + sh-LINC01963. Ten days after injection, DTX solution (10 mg/kg) was intravenously administered to each mouse every 5 days. A total of 24 BALB/c nude mice were divided into four groups (*n* = 6): PC3, PC3 + NC, PC3 + ov-LINC01963, and PC3 + sh-LINC01963. Ten days after injection, DTX solution (1 mg/kg) was intravenously administered to each mouse every 5 days. Tumor size was assessed using a digital caliper. The mice were euthanized, and the subcutaneous tumors were removed. The length and width of the subcutaneous tumors were measured, and the volumes were calculated using the following equation: volume = (length × width)/2. All the mice were included in this study. For the lung metastasis assay, 1 × 10^6^ cells were injected into the tail veins of 6-week-old female BALB/c nude mice. A total of 24 BALB/c nude mice were divided into four groups using the sorting randomization method (*n* = 6): PC3-DR, PC3-DR + NC, PC3-DR + ov-LINC01963, and PC3-DR + sh-LINC01963. Ten days after injection, DTX solution (10 mg/kg) was intravenously administered to each mouse every 5 days. A total of 24 BALB/c nude mice were divided into four groups (*n* = 6): PC3, PC3 + NC, PC3 + ov-LINC01963, and PC3 + sh-LINC01963 groups. Ten days after injection, DTX solution (1 mg/kg) was intravenously administered to each mouse every 5 days. Six weeks post-injection, the degree of lung metastasis was evaluated in all mice using an in vivo imaging system. All the mice were included in this study. Except for the grouping personnel and the laboratory personnel who injected the cells, the other participants did not know the grouping.

### Binding site assay

LINC01963-binded miRNAs were predicted using StarBase v3^20^ and LncBase^21^. miRNA expression in prostate adenocarcinoma was analyzed using StarBase v3^20^. miR-216b-5p binding target genes were predicted using TargetScan 7.2^22^ and miRWalk^23^. Wild-type (WT) or mutant (MUT) LINC01963 and the 3′-UTR of NFAT5 were synthesized by GENEWIZ (Suzhou, China) and cloned into the 3′ end of the firefly luciferase gene in the pmirGLO vector (named WT-LINC01963, MUT-LINC01963, WT-NFAT5, MUT-NFAT5). Then, 30 ng of plasmids were co-transfected into PC3-DR cells with 50 nM miR-216b-5p mimics. After 48 h, luciferase activity was measured using a dual luciferase assay kit (Promega). Renilla luciferase activity was normalized to firefly luciferase activity. RIPA was performed using an RNA immunoprecipitation kit (GENESEED, Guangzhou, China). The purified RNA was analyzed by RT-qPCR to measure LINC01963 and miR-216b-5p expression. The pull-down assay was performed using the PureBinding RNA-Protein pull-down Kit (GENESEED). PC3-DR cells were transfected with a biotinylated LINC01963 probe (Bio-LINC01963) or a negative control probe (Bio-NC) and harvested at 48 h to determine LINC01963 and miR-216b-5p expression via RT-qPCR.

### Western blot

Cells were collected, lysed, and subjected to sodium dodecyl sulfate-polyacrylamide gel electrophoresis. The resolved proteins were transferred to a polyvinylidene fluoride membrane. The membrane was blocked and incubated for 4 h at 25 °C with the following primary antibodies: anti-TrkB (cat: ab134155, 92 kDa 1:1000 [v/v]; Abcam, Cambridge, MA, USA) and anti-GAPDH (cat: ab181602, 1:10,000 [v/v]; Abcam). The membrane was washed at 25 °C for 4 h and incubated with horseradish peroxidase-conjugated secondary antibodies (1:20000 (v/v); Abcam) for 2 h at 25 °C. Protein bands were detected using a chemiluminescent peroxidase substrate (ECL; Amersham, Beijing, China).

### Statistical analysis

The experimental data were analyzed using SPSS 19.0 statistical software (IBM, Inc.). Data are presented as the mean ± standard deviation (SD). One-way analysis of variance was performed to analyze the statistical difference between more than three groups, followed by Tukey’s post-hoc test. A *t*-test was used to analyze the statistical differences between the two groups. Statistical significance was set at *P* < 0.05.

## Results

### LINC01963 expression is upregulated in PC3-DR cells

**Fig. 1:**
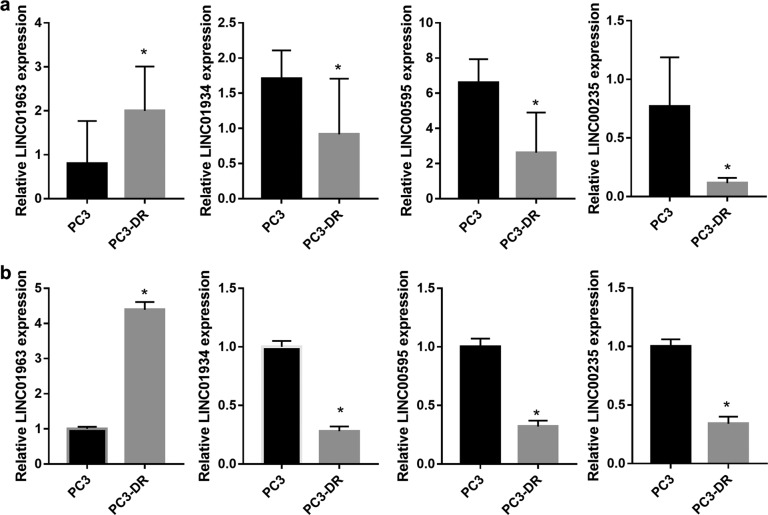
The expression of four lncRNAs in PC3 and PC3-DR cells. PC3 and PC3-DR cells were not treated with DTX in those studies. **a** The expression of four lncRNAs in PC3 and PC3-DR cells is shown according to the results of lncRNA sequencing. **b** The expression of four lncRNAs in PC3 and PC3-DR cells was analyzed by qRT-PCR. Data are shown as the mean ± standard deviation of three technical replicates. *P < 0.05.

PC3 cells had an IC50 of 16.23 nmol/L and PC3-DR cells had an IC50 of 337.1 nmol/L after 48 h of DTX treatment (Supplementary Fig. 1a and 1b). This result shows that PC3-DR cells had higher DTX resistance (20.73-fold change) than PC3 cells. The lncRNA sequencing demonstrated that compared with PC cells, 10,685 lncRNAs were upregulated while 19,949 were downregulated in PC3-DR cells (Supplementary Fig. 1c, Supplementary Table 1). All lncRNA sequencing data were uploaded to GEO (https://www.ncbi.nlm.nih.gov/geo/query/acc.cgi?acc=GSE190648). Moreover, four large intergenic noncoding RNAs (LINC01963, LINC01934, LINC00595, and LINC00235) were selected to verify the sequencing results (Fig. 1). The results showed that LINC01963 expression was upregulated and LINC01934, LINC00595, and LINC00235 expression was lower in PC3-DR cells than in PC3 cells, similar to the results of lncRNA sequencing. The differential expression of LINC01963 was greater than that of the other three lncRNAs; hence, LINC01963 was selected for follow-up research.

### Silenced LINC01963 reduced PC3-DR cell proliferation and metastasis, and enhanced the chemosensitivity of PC3-DR to docetaxel in vitro/in vivo models

**Fig. 2:**
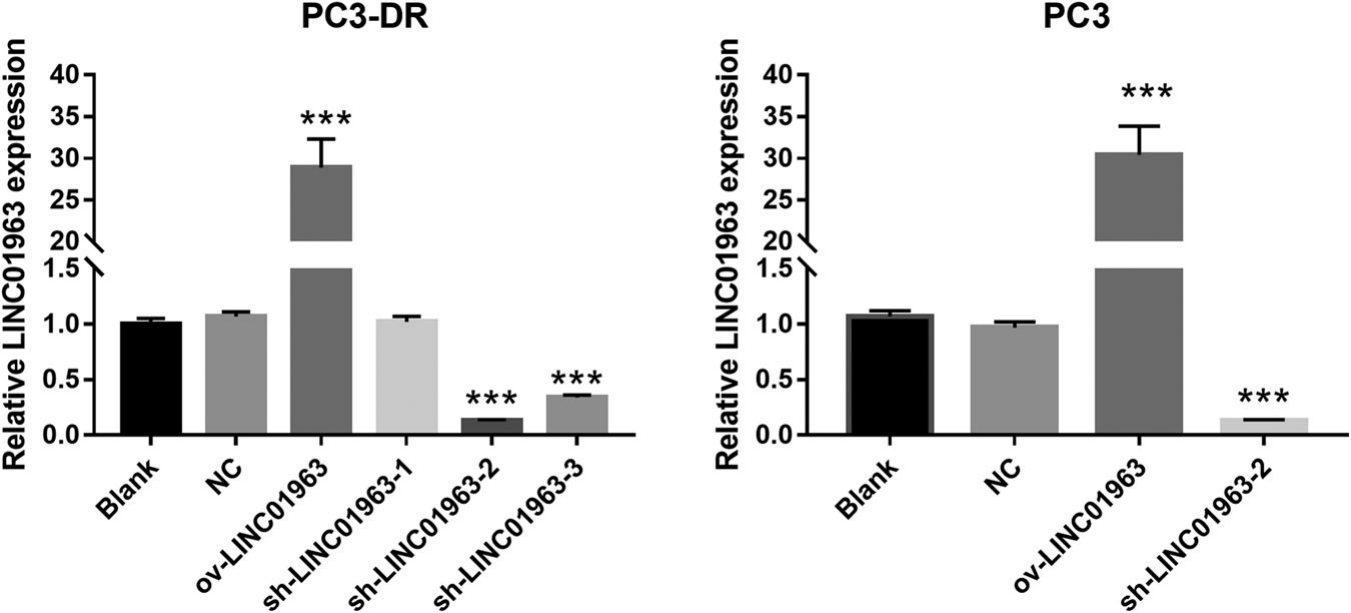
LINC01963 expression was analyzed in PC3 cells and in the PC3-DR stable cell line. PC3 and PC3-DR cells were not treated with DTX in those studies. LINC01963 expression in the PC3-DR and PC3 stable cell line was analyzed by qRT-PCR. Data are shown as the mean ± standard deviation of three technical replicates. shRNA: short hairpin RNA, ov: ovexpression, NC: empty control lentivirus. ***P < 0.001 vs. NC group.

**Fig. 3:**
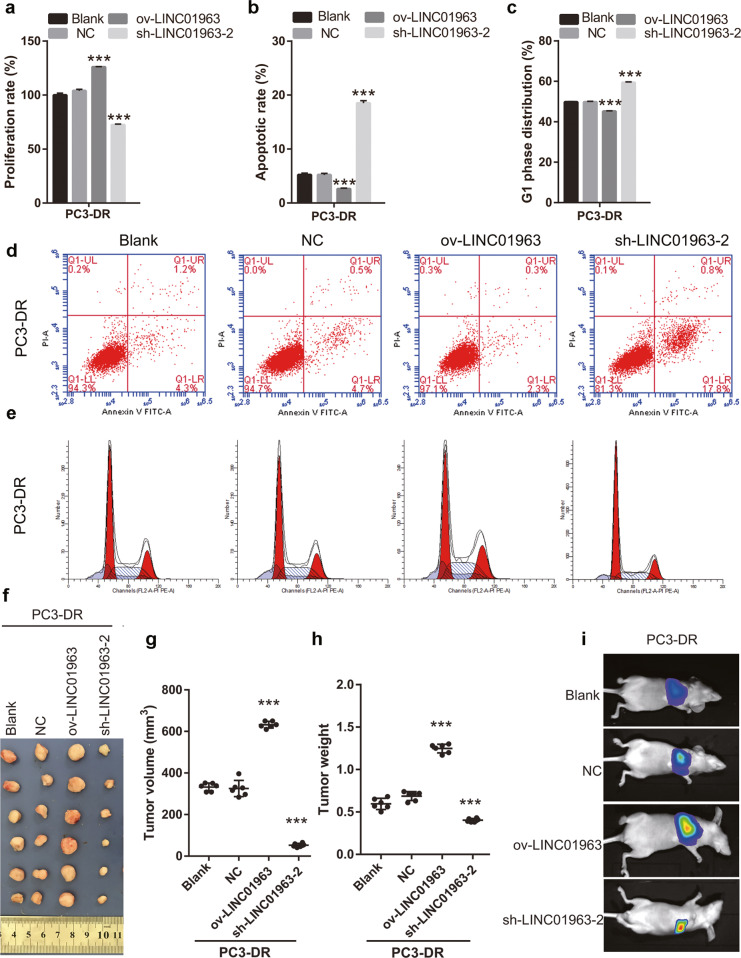
LINC01963 silencing reduced PC3-DR cell survival while LINC01963 overexpression increased PC3-DR cell survival. PC3-DR cells were treated with 40 nmol/L DTX in those studies. **a** Proliferation of PC3-DR cells was analyzed by MTS. **b**, **d** PC3-DR cell apoptosis was analyzed by flow cytometry. Apoptosis is shown as the mean ± SD (**b**), and a representative image of apoptosis is shown (**d**). **c**, **e** The cell cycle of PC3-DR was analyzed by flow cytometry. G1 phase distribution is shown as the mean ± standard deviation of three technical replicates (**c**) and a representative image of the cell cycle is shown (**e**). **f** Tumor tissues in nude mice were excised and photographed 30 days after xenograft study. **g**, **h** Tumor volumes and weights were detected. **i** The degree of lung metastasis in nude mice was evaluated by an in vivo imaging system. ***P < 0.001 vs. NC group.

For in vitro study, PC3-DR cells were infected with ov-LINC01963 and sh-LINC01963-2 lentivirus, and then treated with 40 nM DTX. Compared with NC group, LINC01963 expression was significantly upregulated after infection with ov-LINC01963 lentivirus, while it was significantly downregulated after infection with sh-LINC01963-2 lentivirus in PC3-DR cells (Fig. 2). In addition, the proliferation rate and the migratory and invading cells in the ov-LINC01963 group increased significantly, while that in the sh-LINC01963-2 group decreased significantly compared to that in the NC group in PC3-DR cells (Fig. 3a and Supplementary Fig. 2). Compared with the NC group, the percentage of cells in the G1 phase and apoptosis rate in the ov-LINC01963 group significantly decreased, while those in the sh-LINC01963-2 group significantly increased in PC3-DR cells (Fig. 3b–e). For in vivo study, the tumor volume and weight and the lung metastasis increased significantly in the ov-LINC01963 group, while the tumor volume and weight in the sh-LINC01963-2 group decreased significantly after 40 nmol/L DTX treatment compared to the NC group (Fig. 3f–i). Interference with LINC01963 enhanced the chemosensitivity of PC3-DR to DTX according to the results of in vitro and in vivo experiments. LINC01963 overexpression had the opposite effect.

### LINC01963 overexpression increased PC3 cell proliferation and metastasis, and enhanced the chemoresistance of PC3 cells to docetaxel in vitro/in vivo models

**Fig. 4:**
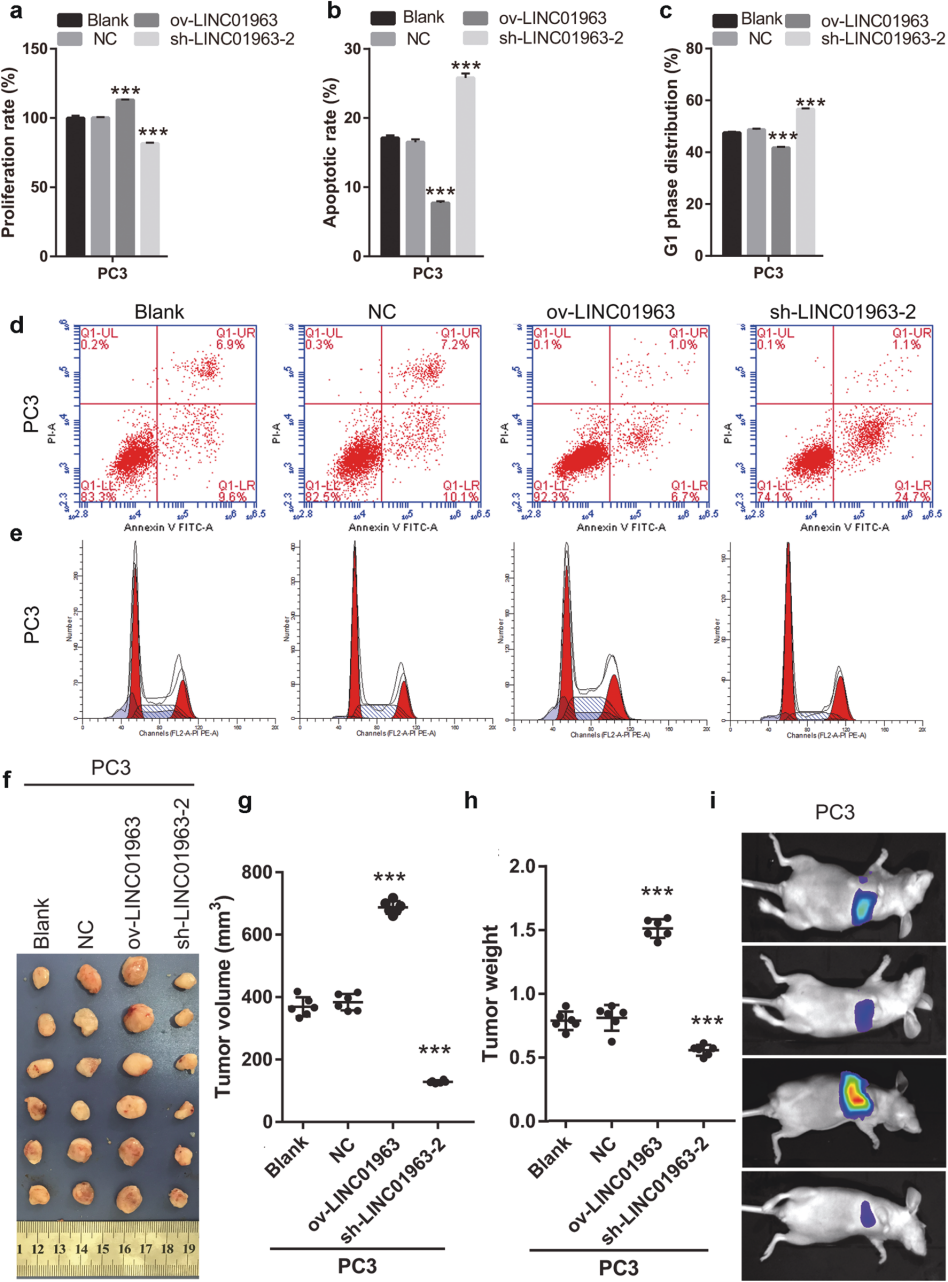
LINC01963 overexpression increased PC3 cell survival while LINC01963 silencing reduced PC3 cell survival. PC3 cells were treated with 4 nmol/L DTX in those studies. **a** Proliferation of PC3 cells was analyzed by MTS. **b**, **d** PC3 cell apoptosis was analyzed by flow cytometry. Apoptosis is shown as the mean ± standard deviation of three technical replicates (**b**), and a representative image of apoptosis is shown (**d**). **c**, **e** The cell cycle of PC3 was analyzed by flow cytometry. G1 phase distribution is shown as the mean  ± SD (**c**) and a representative image of the cell cycle is shown (**e**). **f** Tumor tissues in nude mice were excised and photographed 30 days after xenograft study. **g**, **h** Tumor volumes and weights were detected. **i** The degree of lung metastasis in nude mice was evaluated by an in vivo imaging system. ***P < 0.001 vs. NC group.

Next, for in vitro study, PC3 cells were infected with ov-LINC01963 and sh-LINC01963-2 lentivirus, and PC3-DR cells were subsequently treated with 4 nmol/L DTX. Compared with NC group, LINC01963 expression was significantly upregulated in PC3 cells after infection with ov-LINC01963 lentivirus, while it was significantly downregulated in PC3 cells after infection with sh-LINC01963-2 lentivirus (Fig. 2). Compared to the NC group, the proliferation rate and the migratory and invading cells in the ov-LINC01963 group increased significantly, while that in the sh-LINC01963-2 group decreased significantly in PC3 cells (Fig. 4a and Supplementary Fig. 2). The percentage of cells in the G1 phase and apoptosis rate in the ov-LINC01963 group significantly decreased, while those in the sh-LINC01963-2 group significantly increased in PC3 cells, compared to those in the NC group (Fig. 4b–e). For in vivo study, compared to that in the NC group, the tumor volume and weight and the lung metastasis increased significantly in the ov-LINC01963 group, while the tumor volume and weight in the sh-LINC01963-2 group decreased significantly after 4 nmol/L DTX treatment (Fig. 4f–i). LINC01963 overexpression enhances the chemoresistance of PC3 cells to DTX according to the results of in vitro and in vivo experiments. Interference with LINC01963 had the opposite effect.

### LINC01963 can bind to miR-216b-5p

**Fig. 5:**
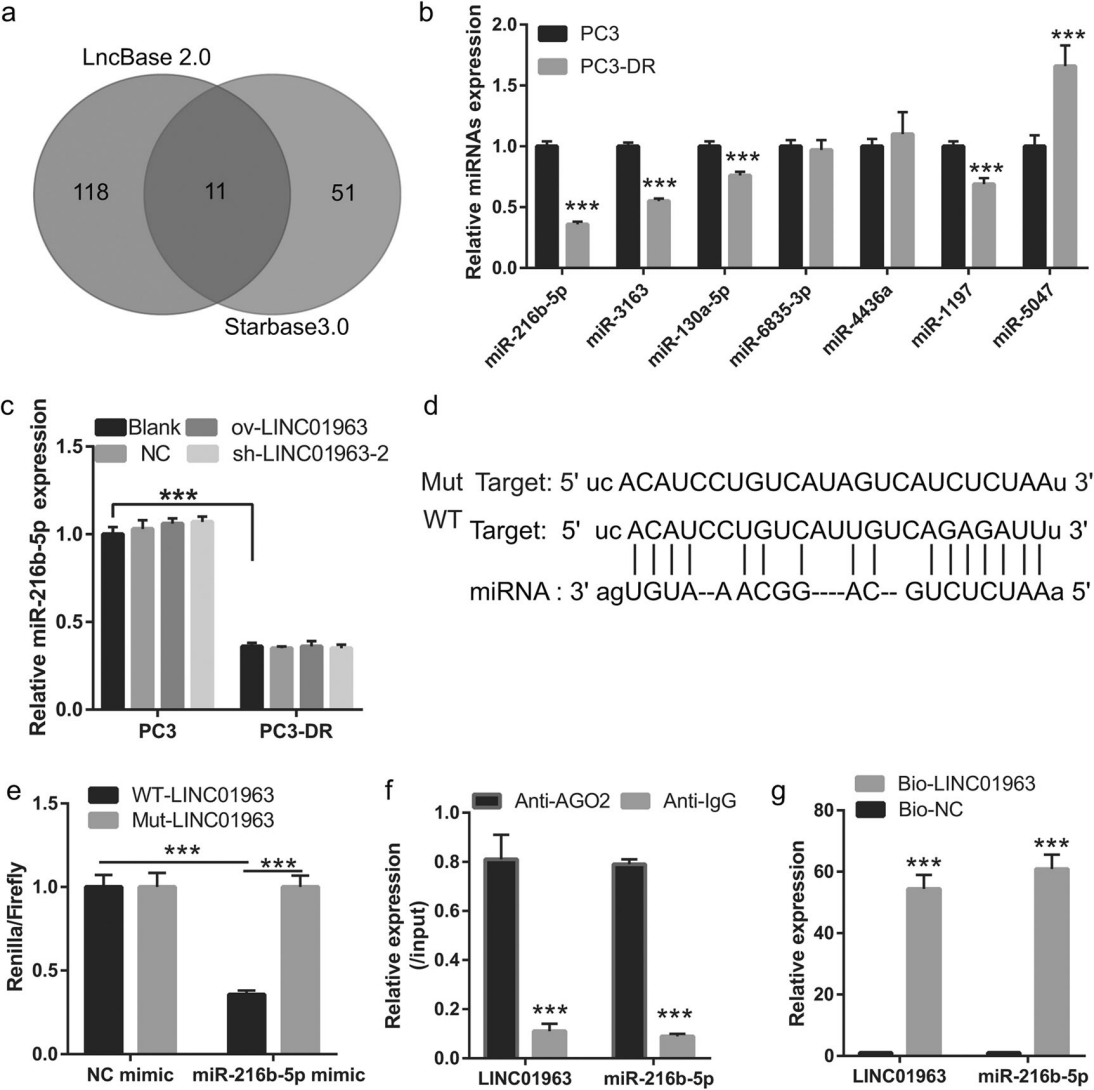
miR-216b-5p was bound by LINC01963. **a** StarBase 3.0 and LncBase 2.0 analysis found that 11 common miRNAs had a promising binding site with LINC01963. **b** miRNA expression was measured by qRT-PCR in PC3 and PC3-DR cells. PC3 and PC3-DR cells were not treated with DTX in those studies. **c** miR-216b-5p expression in the Blank, NC, ov-LINC01963, sh-LINC01963 groups was measured by qRT-PCR in PC3 and PC3-DR cells. PC3 cells were treated with 4 nmol/L DTX and PC3-DR cells were treated with 40 nmol/L DTX. **d** The binding sites between LINC01963 and miR-216b-5p are shown. **e** The binding sites between LINC01963 and miR-216b-5p were determined using a dual luciferase assay. **f** The binding sites between LINC01963 and miR-216b-5p were determined using an AGO-RIPA assay. **g** The binding sites between LINC01963 and miR-216b-5p were determined using a pull-down assay. Data are shown as the mean ± standard deviation of three technical replicates. ***P < 0.001.

StarBase 3.0 and LncBase 2.0 revealed that 11 miRNAs had a promising binding site with LINC01963 (Fig. 5a). In addition, StarBase v3 analysis revealed that 4 out of 11 miRNAs were highly expressed and therefore excluded. Among the remaining seven miRNAs, only miR-216b-5p, miR-3163, miR-130a-5p, and miR-1197 were significantly less expressed in PC3-DR cells than that in PC3 cells, especially miR-216b-5p (Fig. 5b). miR-216b-5p was chosen for further study. miR-216b-5p expression was not significantly different between the NC, ov-LINC01963, and sh-LINC01963 groups in PC3 and PC3-DR cells after DTX treatment (Fig. 5c), suggesting that LINC01963 had no effect on miR-216b-5p expression. The binding sites between LINC01963 and miR-216b-5p are shown in Fig. 5d. The dual luciferase assay results indicated that miR-216b-5p mimics markedly inhibited the luciferase activity of WT-LINC01963 but did not affect the luciferase activity of Mut-LINC01963 (Fig. 5e). Furthermore, RIPA results showed that LINC01963 and miR-216b-5p expression in the anti-AGO2 group was higher than that in the anti-IgG group (Fig. 5f). In addition, compared with the Bio-NC group, LINC01963 and miR-216b-5p expression was significantly enhanced in the Bio-LINC01963 group (Fig. 5g). These findings demonstrate that miR-216b-5p is a target of LINC01963 in PC3-DR cells.

### Silenced miR-216b-5p enhanced DTX resistance of prostate cancer and reverses the effect of interference with LINC01963

**Fig. 6:**
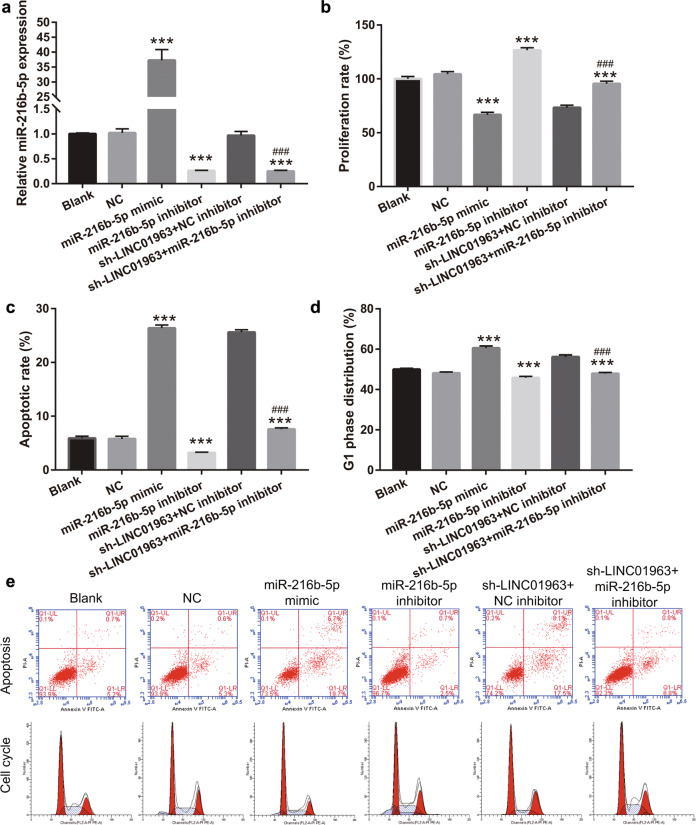
miR-216b-5p reduced DTX resistance of prostate cancer and reversed the effect of LINC01963 on cell proliferation. **a** miR-216b-5p expression in PC3-DR cells was analyzed by qRT-PCR. PC3-DR cells were not treated DTX. **b** Transfected-PC3-DR cell proliferation was analyzed by MTS after treatment with 40 nmol/L DTX. **c**, **d** The apoptosis (**c**) and G1 phase distribution (**d**) of transfected-PC3-DR cells after treatment with 40 nmol/L DTX is shown. Results are shown as the mean ± SD. **e** The apoptosis and cell cycle were analyzed by flow cytometry, and a representative image of apoptosis and the cell cycle are shown. Data are shown as the mean ± standard deviation of three technical replicates. ***P < 0.001 vs. Blank group. ^###^*P* < 0.001 vs. sh-LINC01963-2+NC inhibitor group.

Next, we found that compared with the NC group, miR-216b-5p expression was significantly upregulated and downregulated in PC3-DR cells after transfection with miR-216b-5p mimic and inhibitor, respectively (Fig. 6a). Then PC3-DR cells were treated with 40 nmol/L DTX. Compared to the NC group, the proliferation rate, migration, and invasion in the miR-216b-5p mimic group were significantly decreased, while those in the miR-216b-5p inhibitor group were significantly increased (Fig. 6b, Supplementary Fig. 3). Furthermore, compared to the NC group, the percentage of cells in the G1 phase and apoptosis rate in the miR-216b-5p mimic group significantly increased, while those in the miR-216b-5p inhibitor group significantly decreased (Fig. 6c–e). The above results show that miR-216b-5p overexpression reduced DTX resistance in PC3-DR cells. miR-216b-5p silencing had the opposite effect.

In addition, compared with the sh-LINC01963-2+NC inhibitor group, miR-216b-5p expression was significantly downregulated in the sh-LINC01963-2+ miR-216b-5p inhibitor group in PC3-DR cells (Fig. 6a). Further research showed that compared with the sh-LINC01963-2+NC inhibitor group, the proliferation rate, migration, and invasion were significantly increased, whereas the percentage of cells in the G1 phase and apoptosis rate were significantly decreased in the sh-LINC01963-2+ miR-216b-5p inhibitor group (Fig. 6, Supplementary Fig. 3). These results indicate that miR-216b-5p silencing can reverse the effect of LINC01963 interference on DTX resistance.

### NTRK2 is the target gene of miR-216b-5p

**Fig. 7:**
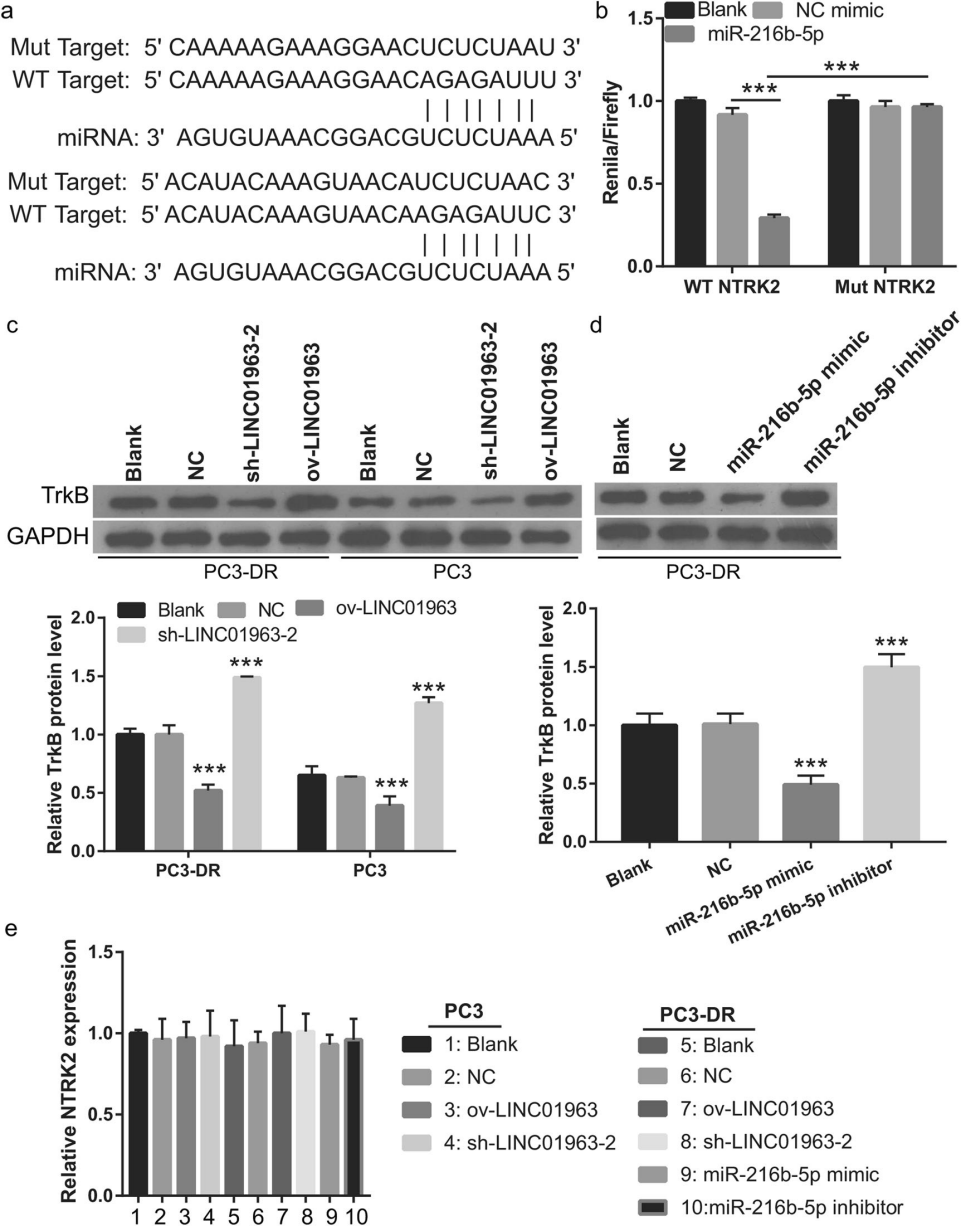
NTRK2 is the target gene of miR-216b-5p. PC3-DR cells were treated with 40 nmol/L DTX and PC3 cells were treated with 4 nmol/L DTX in those studies. **a** The binding sites between the 3′-UTR of NTRK2 and miR-216b-5p were analyzed by Targetscan 7.2 and miRWalk. **b** The binding sites between the 3′-UTR of NTRK2 and miR-216b-5p were determined using a dual luciferase assay. **c** TrkB protein level was analyzed by western blot assay in PC3 cells treated with 4 nmol/L DTX and PC3-DR cells treated with 40 nmol/L DTX. ***P < 0.001 vs. Blank group. **d** TrkB protein level was analyzed by western blot assay in PC3-DR cells treated with 40 nmol/L DTX. ***P < 0.001 vs. Blank group. **e** NTRK2 mRNA expression was measured by qRT-PCR in PC3 cells treated with 4 nmol/L DTX and PC3-DR cells treated with 40 nmol/L DTX. Data are shown as the mean ± standard deviation of three technical replicates.

Targetscan 7.2 and miRWalk found that NTRK2, which encodes the TrkB protein, is the downstream target gene of miR-216b-5p. The binding sites between the 3′-UTR of NTRK2 and miR-216b-5p are shown in Fig. 7a. The dual luciferase assay results indicated that miR-216b-5p mimics markedly inhibited the luciferase activity of WT-3-UTR NTRK2 but did not affect the luciferase activity of Mut-3 NTRK2 (Fig. 7b). Then the TrkB mRNA expression and protein levels were measured in PC3-DR cells treated with 40 nmol/L DTX and PC3 cells treated with 4 nmol/L DTX. Compared with blank group, TrkB protein levels in the NC group were not significantly different in the PC3-DR or PC3 cells. Compared with NC group, TrkB protein levels in the ov-LINC01963 group were significantly increased, while they were significantly reduced in the sh-LINC01963 group in PC3-DR and PC3 cells (Fig. 7c). Moreover, compared with NC group, the TrkB protein level was significantly reduced in the miR-216b-5p mimic group while significantly enhanced in the miR-216b-5p inhibitor group in PC3-DR cells (Fig. 7d). However, NTRK2 expression in all groups did not significantly change in PC3 and PC3-DR cells (Fig. 7e). These findings demonstrate that NTRK2 is a target of miR-216b-5p in PC3-DR cells and that miR-216b-5p regulates TrkB protein levels in post-transcriptional regulation.

### TrkB enhances DTX resistance of prostate cancer and reverses the effect of LINC01963 silencing and miR-216b-5p overexpression

Moreover, compared with ov-NC group, NTRK2 mRNA expression and TrkB levels were significantly upregulated in PC3-DR cells after co-transfection with NTRK2-pcDNA3.1 (ov-NTRK2) and sh-LINC01963 lentivirus or miR-216b-5p mimic (Supplementary Fig. 4a and b). After treatment with 40 nmol/L DTX, the proliferation rate, migration, and invasion in the ov-NTRK2 group were significantly higher than those in the ov-NC group (Supplementary Fig. 4c–e). These results suggest that in PC3-DR cells treated with 40 nmol/L DTX, TrkB enhanced DTX resistance in prostate cancer and reversed the effect of LINC01963 silencing and miR-216b-5p overexpression.

## Discussion

Most patients with prostate cancer eventually develop DTX resistance, which leads to tumor progression or death. Numerous lncRNAs affect DTX chemosensitivity, suggesting that lncRNAs can serve as targets for drug resistance in prostate cancer progression^12,24^. In this study, we found that LINC01963 was overexpressed in DTX-resistant PC3 cells according to our lncRNA sequencing and qRT-PCR results, suggest that it may be a potentially regulatory gene for DTX response in prostate cancer.

Previous studies have shown that LINC01963 expression which is lower in pancreatic carcinoma and oral and oropharyngeal squamous cell carcinoma tissues plays an oncogenic role and acts as a marker of poor prognosis^25,26^. We found that LINC01963 silencing enhanced the chemosensitivity of PC3-DR cells to DTX, indicating that LINC01963 is a potential therapeutic target for treating patients with prostate cancer with DTX resistance. Previous studies have shown that LINC01963 overexpression can inhibit the growth and metastasis of pancreatic cancer^26^, and in this study, we found that LINC01963 overexpression promotes chemotherapy resistance in prostate cancer, suggesting that LINC01963 plays different roles in different tumors.

miRNAs also play an important role in DTX resistance in prostate cancer^27,28^. In this study, miR-216b-5p expression was significantly lower in PC3-DR cells than in PC3 cells. Previous study found that miR-216b-5p acts as an anticancer gene in pancreatic cancer and hepatocellular carcinoma^29,30^. miR-216b-5p was a key gene in enhancing the paclitaxel sensitivity of ovarian cancer cells^31^. In addition, miR-216b-5p overexpression inhibits prostate cancer cell proliferation and migration and enhances the chemosensitivity of prostate cancer cells to paclitaxel^32,33^. These studies suggest that miR-216b-5p can inhibit prostate cancer development and paclitaxel resistance. Similar to these previous results, miR-216b-5p inhibits progression and improved DTX resistance in prostate cancer. Meanwhile, miR-216b-5p was bound by SNHG1 and LINC00518 which has been shown to regulate cancer cells chemoresistance^31,33^. In this study, we found that silencing LINC01963 enhanced the chemosensitivity of PC3-DR to DTX by binding to miR-216b-5p.

Next, we found that miR-216b-5p can bind to the 3′-UTR of NTRK2 and inhibit TrkB protein levels. TrkB stimulates tumor cell survival and angiogenesis and contributes to chemotherapy resistance and tumorigenesis, suggested that TrkB is a novel target for improving chemotherapy resistance and tumorigenesis^34,35^. TrkB protein levels are reportedly overexpressed in prostate cancer tissues, and enhanced TrkB expression facilitates tumor growth and metastasis^36^. In this study, we found that TrkB enhanced DTX resistance in prostate cancer and reversed the effects of LINC01963 silencing and miR-216b-5p overexpression. In agreement with previous results, this study suggested that TrkB contributes to chemotherapy resistance, tumorigenesis, and metastasis^34–36^. These findings also suggested that LINC01963 silencing inhibited TrkB protein level to enhance the chemosensitivity of PC3-DR cells to DTX by binding to miR-216b-5p.

In conclusion, the silencing of the LINC01963/miR-216b-5p/TrkB axis can enhance the chemosensitivity of PC3-DR cells to DTX. These results illustrate the mechanism of DTX resistance in prostate cancer and suggest that LINC01963 is a novel therapeutic target for treating prostate cancer patients with DTX resistance.

## Supplementary information

Supplementary Table 1

Supplementary Figures

Original image of western blot

## Data Availability

The data used to support the findings of this study are included in this paper.
