# Supplementary material for: Silencing of LINC01963 enhances the chemosensitivity of prostate cancer cells to docetaxel by targeting the miR-216b-5p/TrkB axis
Source: Lab Invest. 2022 Feb 12;102(6):602–12. doi: 10.1038/s41374-022-00736-4 (PMC9162921; doi:10.1038/s41374-022-00736-4)
Supplement: Supplementary file 2 — Supplementary Figures [file 41374_2022_736_MOESM2_ESM.pdf]

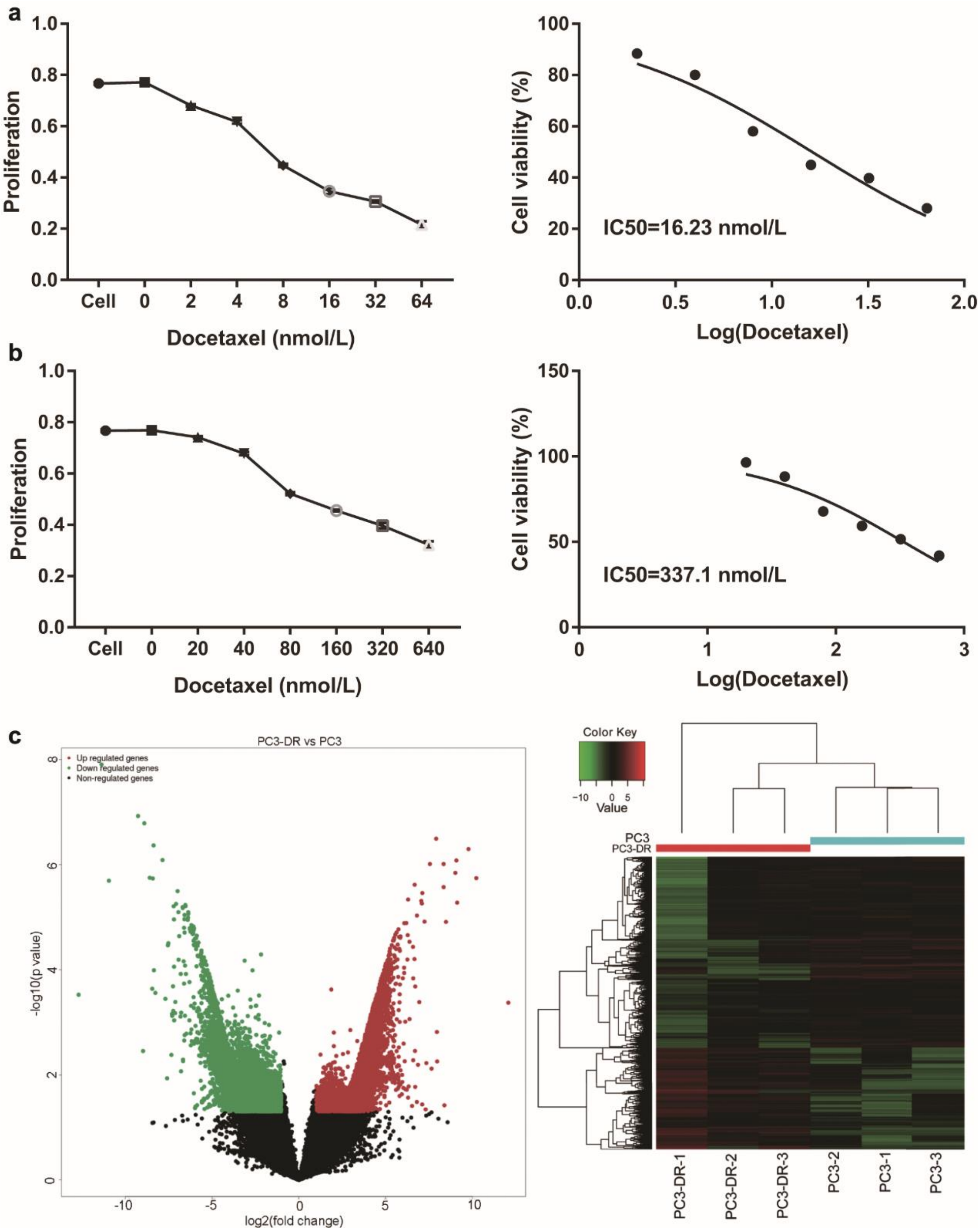

Supplementary Fig. 1 Abnormally expressed lncRNAs between PC3 cells and PC3-DR cells. (a) The effect of DTX on PC3 cell proliferation. (b) The effect of DTX on PC3-DR cell proliferation. (c) Volcano plot (left) and heat map (right). Abnormally expressed lncRNAs were analyzed by lncRNA sequencing analysis in PC3 cells and PC3-DR cell. IC50: The half maximal inhibitory concentration.

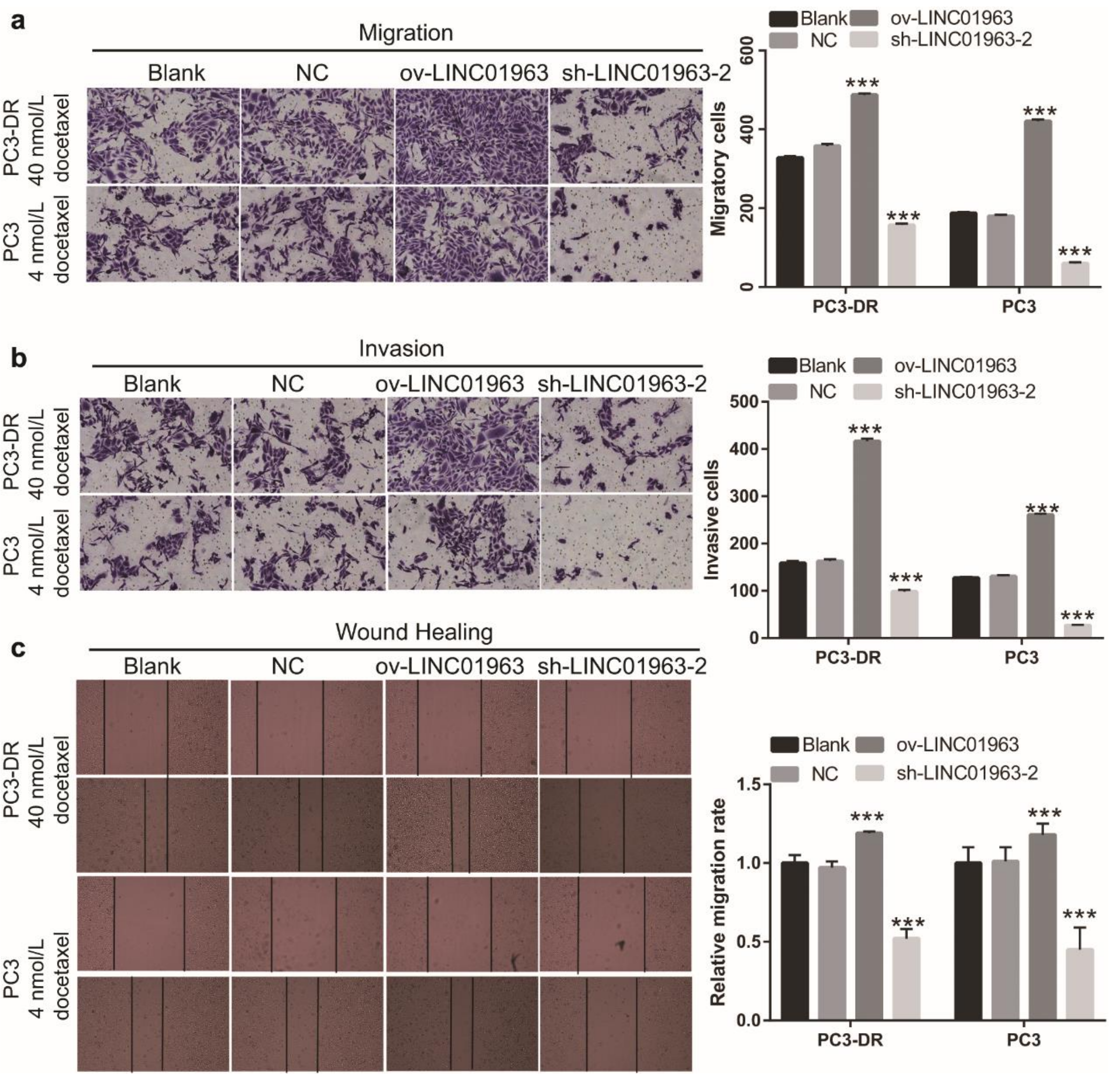

Supplementary Fig. 2 LINC01963 silencing reduced PC3-DR and PC3 cell migration and invasion. PC3-DR cells were treated with 40 nmol/L DTX and PC3 cells were treated with 4 nmol/L DTX in those studies. (a and b) The migration (a) and invasion (b) assays were analyzed by Transwell filters uncoated or precoated with Matrigel (magnification, ×100). (C) The migration assays were analyzed by a wound healing assay. Data are shown as the mean ± standard deviation of three technical replicates. ***P < 0.001 vs. NC group.

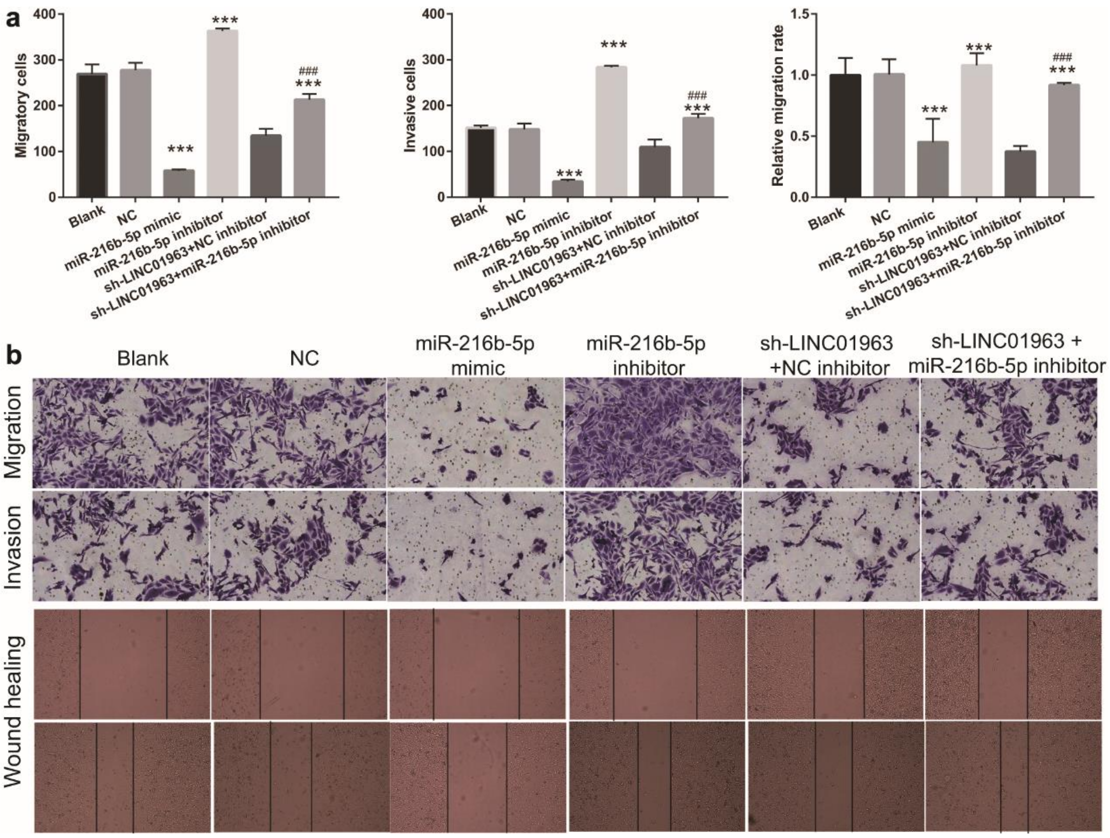

Supplementary Fig. 3 miR-216b-5p reversed the effect of LINC01963 on cell migration and invasion. PC3-DR cells were treated with 40 nmol/L DTX in those studies. (a) Cell migration, invasion, and the relative migration rate are shown as the mean ± SD. (b) The migration and invasion assays were analyzed using Transwell filters uncoated or precoated with Matrigel (magnification, ×100), and the migration assays were analyzed by a wound healing assay. Data are shown as the mean ± standard deviation of three technical replicates. ***P < 0.001 vs. Blank group. ###P < 0.001 vs. sh-LINC01963-2+NC inhibitor group.

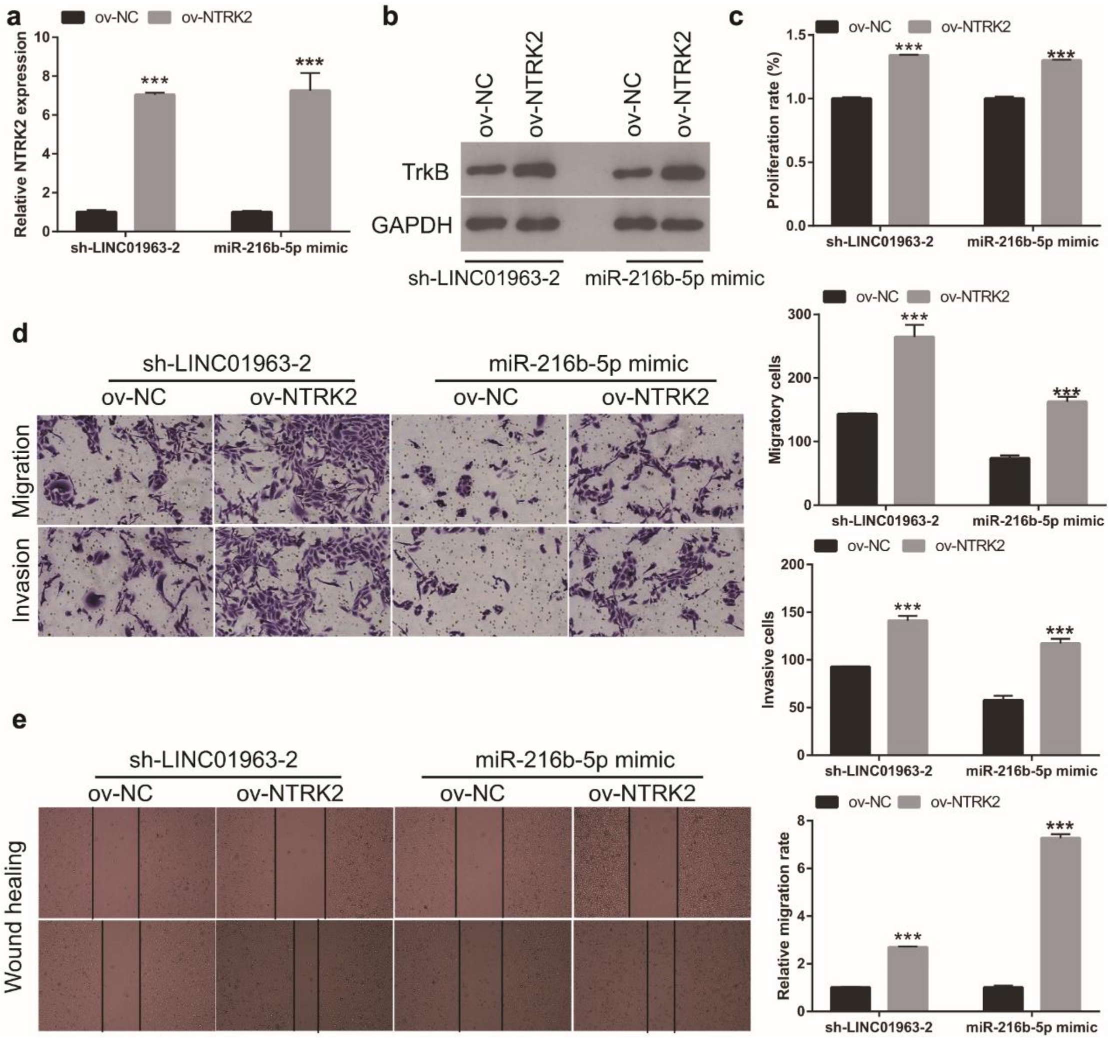

Supplementary Fig. 4 TrkB enhances DTX resistance of prostate cancer. PC3-DR cells were treated with 40 nmol/L DTX in those studies. (a) NTRK2 mRNA expression was measured by qRT-PCR in PC3-DR cells. (b) TrkB protein level was analyzed by western blot assay in PC3-DR cells. (c) PC3-DR cell proliferation was analyzed by MTS. (d) The migration and invasion assays were analyzed using Transwell filters uncoated or precoated with Matrigel (magnification, ×100). The results are shown as the mean ± SD. (e) The migration assays were analyzed by a wound healing assay, and the relative migration rate is shown as the mean ± standard deviation of three technical replicates. ***P < 0.001 vs. ov-NC.
