# Supplementary figures and images for: Silencing of LINC01963 enhances the chemosensitivity of prostate cancer cells to docetaxel by targeting the miR-216b-5p/TrkB axis
Source: Lab Invest. 2022 Feb 12;102(6):602–12. doi: 10.1038/s41374-022-00736-4 (PMC9162921; doi:10.1038/s41374-022-00736-4)

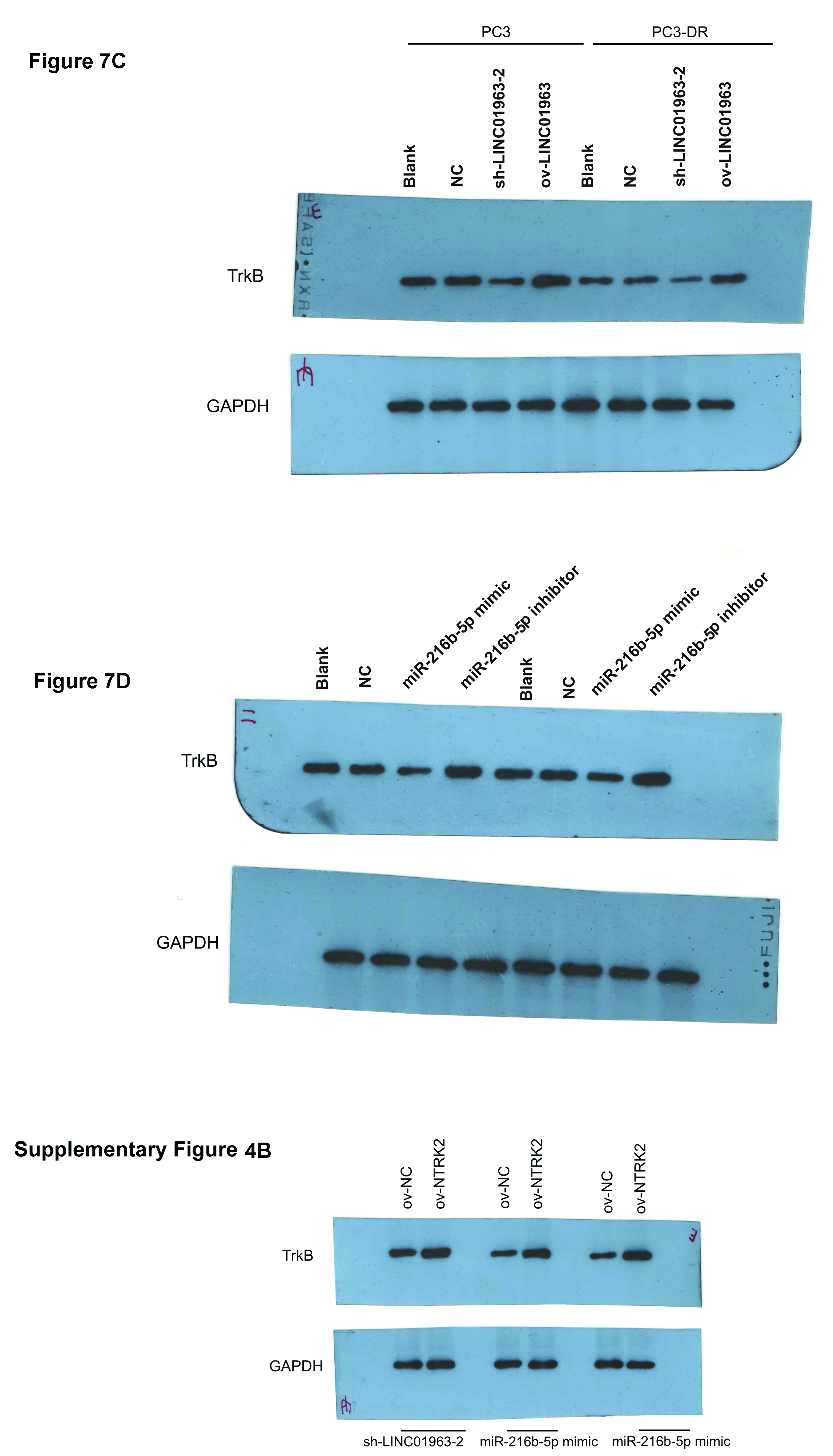

Supplement: Supplementary file 3 — Original image of western blot [file 41374_2022_736_MOESM3_ESM.tif]
